# Early-Stage and Locally Advanced Cervical Cancer during Pregnancy: Clinical Presentation, Diagnosis and Treatment

**DOI:** 10.3390/medicina60101700

**Published:** 2024-10-16

**Authors:** Hanna Mruzek, Joanna Kacperczyk-Bartnik, Anna Dańska-Bidzińska, Michał Ciebiera, Laretta Grabowska-Derlatka, Paweł Derlatka

**Affiliations:** 1 Students’ Scientific Group, II Department of Obstetrics and Gynecology, Medical University of Warsaw, 02-091 Warsaw, Poland; 2 II Department of Obstetrics and Gynecology, Medical University of Warsaw, 02-091 Warsaw, Poland; 3 Second Department of Obstetrics and Gynecology, Centre of Postgraduate Medical Education, 00-189 Warsaw, Poland; 4 Warsaw Institute of Women’s Health, 00-189 Warsaw, Poland; 5 Second Department of Clinical Radiology, Medical University of Warsaw, 02-091 Warsaw, Poland

**Keywords:** cervical dysplasia, cervical intraepithelial neoplasia, cervical cancer, pregnancy, human papillomavirus viruses, cytology

## Abstract

In this comprehensive review supported by clinical examples, the authors explore the topic of cervical cancer in pregnancy, with emphasis on potential pre-cancer progression, the possibility of coexisting preinvasive and invasive disease, and neoadjuvant chemotherapy. This manuscript addresses the challenges of managing cervical cancer in pregnant women with a pregnancy-preserving approach, including the importance of screening, the timing of surgery, and the impact of pregnancy on the course of the disease. The first case study illustrates the potential for a benign cervical lesion to transform into a malignant one during pregnancy and the possible coexistence of preinvasive lesions together with early-stage cervical cancer. It also questions the rationale behind the non-treatment of pregnant patients initially diagnosed with CIN 2/3 during pregnancy. The second presented clinical example shows the histologically confirmed response to neoadjuvant chemotherapy, resulting in a radiologically diagnosed FIGO stage IIA1 being downgraded to adenocarcinoma in situ in the histology report after surgery performed six weeks postpartum. The treatment of cervical cancer, which is becoming increasingly prevalent among pregnant women, and the necessity for an individualized diagnostic and therapeutic approach represent significant challenges for contemporary medicine. Discrepancies in therapeutic options proposed among centers within the same region lead to the conclusion that there is a need for centralization and unification of evidence-based management in referral centers with both high-level oncological and perinatal care.

## 1. Introduction: Etiology and Epidemiology

Cervical cancer is a malignant neoplasm that originates in the uterine cervix. The primary etiological agent is human papillomavirus (HPV), with HPV 16 and 18 being the most prevalent types. The most common histological subtypes are squamous cell carcinoma and adenocarcinoma, accounting for approximately 70% and 25% of all cervical cancers, respectively [1]. It is estimated that the majority of sexually active women will become infected with HPV at some point during their lifetime. However, less than 10% of these infections will persist, increasing the risk of developing cervical cancer. Invasive cervical cancer is preceded by a long phase of preinvasive disease known as cervical intraepithelial neoplasia (CIN). It is divided into three stages: CIN 1 (low-grade squamous intraepithelial lesions, LSILs), 2, and 3 (high-grade squamous intraepithelial lesion, HSILs), corresponding to the degree of cell abnormality in the cervical epithelium [2].

Cervical cancer is the fourth most commonly diagnosed female malignancy and the fourth leading cause of cancer mortality in women worldwide. It is also the most common gynecological cancer detected during pregnancy by established screening strategies. Over the past few decades, with an increase in the average socioeconomic level, the introduction of screening programs, and widespread access to vaccination, a decrease in cervical cancer incidence and mortality rates has been observed. However, the situation appears to be different for pregnant women, in whom a significant increase in the incidence of cervical dysplasia diagnosed during pregnancy has been noted in recent years [3,4,5].

The incidence of cervical cancer in pregnancy is estimated at 1.4–4.6 cases per 100,000 pregnancies [6]. According to population-based studies, the number of cases of cervical cancer during pregnancy will increase. This trend will be particularly noticeable in countries where women choose to become pregnant later and where access to non-invasive prenatal testing to detect asymptomatic cancers will be reimbursed and facilitated [6]. Some studies have indicated that elevated levels of estrogen, progesterone, and human chorionic gonadotropin during pregnancy may be positively correlated with the risk of developing HPV 16 and HPV 18 infection. On the other hand, around 80% of cervical cancer cases detected during pregnancy are stage I disease cases, which permit the continuation of pregnancy and fertility-sparing treatment, and have higher chances of achieving complete remission [7].

Together with the increasing number of cancer cases detected in pregnancy, the management strategies are gradually changing. De Haan et al. published an analysis based on data from the International Network on Cancer, Infertility and Pregnancy (INCIP) registry including 1170 pregnant patients with primary invasive cancer managed between 1996 and 2016 [7]. The results showed that every five years, the likelihood of receiving oncological treatment during pregnancy increased (RR 1.10, 95% CI 1.05–1.15), more livebirths were observed (RR 1.04, 95% CI 1.01–1.06), and fewer iatrogenic preterm deliveries occurred (RR 0.91, 95% CI 0.84–0.98). The prognosis of patients managed for cervical cancer during pregnancy shows no negative impact of pregnancy on the oncological outcome. This is why, in most cases, pregnancy-preserving management is recommended [6,8]. No sufficient data are available regarding the prognosis of patients with nodal metastases (including micrometastases) treated with chemotherapy during pregnancy and delayed chemoradiation postpartum [6].

## 2. Screening during Pregnancy

Screening for cervical cancer during pregnancy is based on the “three-step model” of cytology, colposcopy, and cervical biopsy. Screening pregnant patients with HPV testing or co-testing demonstrates a higher detection rate of HSIL+ changes than cytology alone [9]. If a result suggests an abnormality, it is necessary to deepen the diagnosis by performing colposcopy [8]. It is recommended to collect a cervical smear routinely during pregnancy, preferably as part of the first prenatal visit, unless the previous screening test was performed within the six months before conception. Abnormal cytology is found in approximately 5–8% of pregnant women, while the incidence of CIN in pregnancy is estimated at approximately 1% of the pregnant population [5,10]. Patients with cervical histology indicative of HSIL (CIN2/3) in pregnancy should undergo screening every 12 weeks.

## 3. Progression of Cervical Dysplasia to Invasive Cancer

In pregnancy, a high-grade squamous intraepithelial lesion (HSIL) has an estimated risk of progression to invasive carcinoma of less than 2% [11,12]. Reduced immunity during early pregnancy may suggest that pregnancy could promote cervical cancer progression [13]. However, this claim remains controversial and requires further study. In its preinvasive state and early stages, cervical cancer is often asymptomatic and is usually detected incidentally during routine screening. One of the most common symptoms reported by women is abnormal vaginal bleeding. However, in some women, the first symptom may be excessive vaginal discharge, which may be watery, mucosal, or purulent [14]. In such a situation, when progression occurs before 22 weeks of pregnancy, the recommended treatment is cervical conization. After 22 gestational weeks, it may be appropriate to consider deferring surgical treatment until after delivery. Although surgery is possible in all trimesters of pregnancy, it is best performed at the beginning of the second trimester, when the risk of miscarriage is lower [6].

## 4. Diagnosis

In case of suspected cervical cancer in pregnancy, histological confirmation is required [15]. Society guidelines recommend colposcopy-oriented biopsy without endocervical curettage or small excisional procedures [15]. Referral criteria for colposcopy in pregnant patients are the same as for the general population [16]. Collecting biopsies in pregnant patients seems safe but is recommended only in case of suspected invasion [16]. Published data indicate the safety of excisional procedures between the 15th and 19th gestational weeks; however, they should be performed for the therapeutic indications rather than diagnostic [16,17]. Management in experienced colposcopy units is advised [16].

The use of gadolinium contrast agents in magnetic resonance imaging (MRI) during pregnancy remains a controversial topic. Medical societies do not recommend it because of the potential risk of toxicity [15,18]. Conversely, in a cohort study analyzing 1,424,105 deliveries between 2003 and 2015, exposure to MRI during the first trimester was not associated with increased risk of harm to the fetus or in early childhood [19]. In the same analysis, greater risk of fetal or neonatal death and rheumatological, inflammatory, or infiltrative skin conditions were reported in cases of gadolinium use. As a large cohort study based on data retrieved from registries of historical cohorts, certain limitations of the analysis were identified, including missing information on the indications for MRI. Another limitation was associated with the size of the analyzed subgroups, posing a potential risk of insufficient sample size to support a statistical comparison of contrast MRI (n = 397) vs. non-contrast MRI (n = 1340). Despite these results, exposure to gadolinium contrast agents in utero persisted, mainly in the first weeks of pregnancy before patients were aware of it. A study led by the U.S. Food and Drug Administration (FDA) revealed that the incidence of gadolinium contrast agent use was reported in 1 in 860 pregnancies [20]. A recently published analysis of real-world data based on a database retrieved from Medicaid insurance enabled comparison of the course of pregnancy of 782 women exposed to gadolinium-enhanced MRI and 5209 women who underwent MRI without gadolinium contrast agents [21]. This analysis showed no differences in the subgroups regarding the incidence of fetal or neonatal deaths (1.4% vs. 1.4%, adjusted RR 0.73, 95%CI 0.34–1.55) nor admission to the NICU (adjusted RR 1.03, 95%CI 0.76–1.39). However, due to the gadolinium retention in various tissues, the authors emphasized the need for further evaluation of the potential impact of exposure on the incidence of subacute and chronic adverse outcomes.

## 5. Multidisciplinary Approach

Management of pregnant patients with invasive disease requires a multidisciplinary approach. The team of experts providing care for women with cervical cancer in pregnancy should include gynecological oncologists, neonatologists, obstetricians, pathologists, anesthesiologists, radiation oncologists, medical oncologists, and psycho-oncologists [15]. Treatment in referral centers with both high-level oncological and perinatal care is recommended [15]. Both the oncological safety of the mother and fetal survival without additional morbidity are the aims of treatment plans established by multidisciplinary teams, based on the patient’s intention, tumor stage, and gestational age [15].

## 6. Chemotherapy during Pregnancy

Most chemotherapeutic agents do not cross the placenta [6]. Platinum-based drugs are characterized by substantial placental transfer; however, evidence-based data report the safety of carboplatin during pregnancy [22,23]. A retrospective cohort analysis of the data from the INCIP registry indicated an association between treatment with platinum-based chemotherapy and small-for-gestational-age infants (OR 3.12, 95% CI 1.45–6.70) [7]. In the same study, the authors observed an association between taxane chemotherapy and NICU admission (OR 2.37, 95% CI 1.31–4.28). Initiation of chemotherapy based on cisplatin or carboplatin, in combination with taxanes, can be considered after 14 weeks of pregnancy [15]. Several reports mention fetal ototoxicity associated with cisplatin exposure; therefore, carboplatin is preferred [6]. Administration of bevacizumab and checkpoint inhibitors during pregnancy are contraindicated [15]. In order to avoid myelotoxicity-related adverse events at the time of delivery, the last cycle of chemotherapy should be administered 2–4 weeks before the planned cesarean section [6,15]. The effectiveness of neoadjuvant chemotherapy for cervical cancer in pregnancy on pathologic response was evaluated in a systematic review that included data from 199 patients [24]. Complete response was reported in 72 (36.2%) patients, partial response in 78 (39.2%) patients, in situ carcinoma or high-grade squamous intraepithelial lesion in 22 (11.1%) patients, and progression or no response in 27 (13.6%) patients [24].

## 7. Delivery

The recommended mode of delivery for patients with cervical cancer is cesarean section [15]. Vaginal delivery can cause tumor damage, excessive bleeding, or even the implantation of malignant cells at the perineal incision site. Therefore, if cancer is diagnosed, a cesarean section is advised [6]. Iatrogenic labor before the completion of the 37th week of pregnancy is discouraged to prevent acute complications in the newborn and potential long-term sequelae associated with prematurity [6]. In case of preinvasive cervical lesions, cesarean section is not indicated. A favorable correlation has been found between CIN3 regression and vaginal delivery [25].

## 8. Surgical Treatment

**Figure 1:**
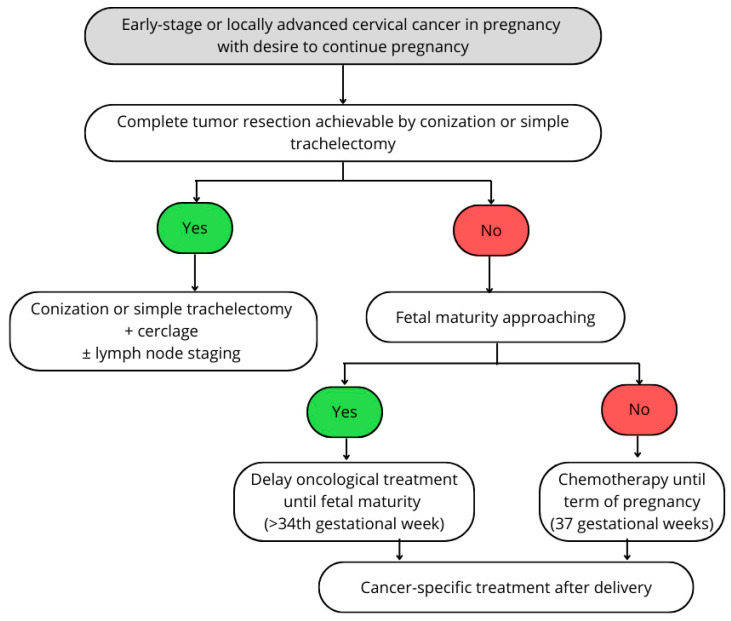
Management of early-stage or locally advanced cervical cancer in pregnancy with desire to continue pregnancy (Figure created based on recommendations from the ESGO/ESTRO/ESP guidelines for the management of patients with cervical cancer—updated in 2023 [15]).

Qualification for surgery depends on disease stage, gestational age, and patients’ fertility intentions (Figure 1) [15]. If laparotomy during pregnancy is considered, scheduling is recommended between the 14th and 16th weeks of gestation [6]. For patients with LVSI-negative IA1 tumors, treatment with cone biopsy can be sufficient [6]. Staging lymphadenectomy is advised for LVSI (+) IA1 and IA2-IB1 disease [6]. Simple trachelectomy and lymph node dissection is an option for patients in the first trimester with stages IA2-IB1 [26]. Performing nodal resection is not recommended after the 22nd to 24th gestational week because of the low number of obtained nodes [6,15]. After the 22nd week of gestation, scheduling surgery after delivery is preferred, with the optional administration of chemotherapy in the second and third trimester [6].

## 9. Sentinel Lymph Node Biopsy

Non-invasive surgical methods for staging lymphadenectomy between the 14th and 16th gestational week are preferred [15]. The use of a sentinel lymph node biopsy with indocyanine green is still considered an experimental method according to the ESGO/ESTRO/ESP guidelines [15]. However, with more evidence available, a gradual shift in practice regarding sentinel lymph node dissection in cervical cancer is being observed. Clinical guidelines by the Spanish Society of Medical Oncology and the Spanish Group for Investigation on Gynecologic Cancer (SEOM-GEICO) published in August 2024 state that sentinel lymph node mapping plays a crucial role in staging early-stage cervical cancer, particularly for FIGO stages IA1 with lymphovascular space invasion, IA2, and IB1 [27]. Sentinel lymph nodes should be assessed using ultrastaging techniques to identify low-volume metastases, while non-sentinel nodes do not require this additional analysis [27]. Similarly, according to the Polish Society of Gynecological Oncology Guidelines from 2024, sentinel lymph node biopsy is the preferred method for assessing lymph node status in tumors below 2 cm in diameter, and it may be considered for tumors larger than 2 cm and up to 4 cm in diameter [28]. Detailed information on the use of sentinel lymph node biopsy in cervical cancer according to different scientific societies is presented in Table 1.

Data from the international, multicenter, prospective, single-arm SENTIX trial (NCT02494063) showed that two-year disease-free survival and overall survival in patients with early-stage cervical cancer after sentinel lymph node biopsy were comparable with those after pelvic lymphadenectomy reported in the published literature [29]. Based on these findings presented in 2024, Cibula et al. concluded that sentinel lymph node biopsy with ultrastaging without further lymphadenectomy is not associated with an increased risk of recurrence [29,30]. These practice-changing study results could be especially helpful for the management of the population of pregnant patients with cervical cancer who require minimal reasonably achievable intervention to minimize the potential risks to the fetus.

The transfer of indocyanine green across the human placenta is minimal [31]. This limited maternal-to-fetal transfer is likely due to the involvement of placental uptake carriers from the organic anion-transporting polypeptide family. Under normal circumstances, the placenta acts as an effective barrier, preventing significant passage of indocyanine green [31]. However, because reproductive studies in animals have not been performed with indocyanine green, and it was not explored whether the drug could cause harm to a fetus when administered to a pregnant woman or impact reproductive capacity, indocyanine green should only be used in pregnancy if its necessity is clearly established [32].

## 10. Adjuvant Treatment

Following the updated ESGO/ESTRO/ESP guidelines published in 2023, image-guided brachytherapy is recommended as an adjuvant treatment after surgery in cases of well-defined, localized areas at high risk of local recurrence, e.g., with positive resection margins in the vagina or parametrium [15]. The recognition of strict criteria for adjuvant radiotherapy is further supported by recently published results of the SCCAN study subanalyses [33]. The reported data showed that in patients with intermediate-risk, early-stage cervical cancer, radical surgery alone demonstrated equivalent disease-free and overall survival rates compared to the combination of radical surgery and adjuvant (chemo)radiotherapy [33]. Previously, adjuvant radiotherapy after surgery with radical intent was considered in the presence of a combination of risk factors at final pathology, e.g., tumor size, LVSI, and depth of stromal invasion [34]. Before initiating radiotherapy or chemoradiation, patients with functional gonads should be informed about options for preserving their gametes, embryos, or ovarian tissue, in case they wish to attempt conception in the future through assisted reproductive technology, potentially with the support of a gestational carrier [35,36].

## 11. Case Study I: High-Grade Squamous Intraepithelial Lesions and Progression to Invasive Cancer or Coexistence of Preinvasive Lesions and Early-Stage Cervical Cancer during Pregnancy

A 29-year-old primiparous woman underwent a Pap smear at the 11th gestational week. In accordance with the 2014 Bethesda system classification, the cervical smear examination revealed the presence of high-grade squamous intraepithelial lesion (HSIL), indicating moderate to severe dysplasia, CIN 2/CIN 3. The result of a Pap smear conducted two years earlier revealed no abnormalities. The patient had no pre-existing chronic diseases. She had previously used oral contraceptives for a period of four years. After her decision to become pregnant and a year of unsuccessful attempts, ovulation induction was performed. The patient had no previous history of childbirth or miscarriage.

Following the abnormal Pap smear result, a cervical biopsy was performed. At the 15th gestational week, the patient presented to the Emergency Unit with severe lower abdominal pain, constipation, and profuse vaginal discharge, without any dysuric symptoms. A culture was performed to exclude infection, which was negative, and a colposcopy was performed, during which an ectopically altered cervical disk was noted. An ultrasound examination of the fetus and uterus revealed no abnormalities. The histopathological examination of the cervical biopsy result was CIN 3. Considering the results of the examination and the patient’s reproductive intentions, a cervical conization procedure was scheduled for the postpartum period. During the pregnancy, this patient was diagnosed with preinvasive lesions; however, considering the final histology result obtained after postpartum conization, two clinical scenarios are possible. The first one includes progression to invasive disease and the second one suggests the coexistence of preinvasive and invasive lesions, with the detection of CIN3 changes in collected biopsies.

At the end of the second trimester, at the 25th gestational week, a repeat biopsy was performed. The specimen was evaluated as showing non-invasive high-grade squamous cell carcinoma. The depth of infiltration was less than 1.7 mm, without lymph-vascular space invasion. A gadolinium-enhanced MRI was conducted, which showed no pathological masses in the vagina, uterus, or adjacent areas.

The delivery was scheduled at 38 weeks via cesarean section, which was uncomplicated. The neonate, of normal height and weight, received an Apgar score of 10 and showed no abnormalities on general examination and transcranial ultrasound. Following the cesarean section, the patient was again referred for an MRI scan, which demonstrated no evidence of cervical infiltration.

Two weeks later, the patient was admitted for a scheduled surgical conization. The procedure commenced with a cervical amputation, followed by a laparoscopic sentinel lymph node procedure. Upon visual inspection of the uterine body, adnexa, abdominal organs, and retroperitoneal lymph nodes, no macroscopic changes were identified. The vaginal portion of the cervix was then subjected to surgical conization. The collected material was histopathologically evaluated, which confirmed the diagnosis of high-grade squamous cell carcinoma, measuring 1.2 × 0.7 cm. This was classified as IB1 according to the FIGO 2018 classification, which defines invasive carcinoma with a diameter of no more than 2 cm and a depth of infiltration of the lining more than 5 mm. Additionally, an examination of the sentinel iliac lymph nodes was performed, which did not reveal the presence of metastases. Scrapings from the cervical canal revealed the presence of necrotic tissue, accompanied by chronic inflammation. Upon analysis of the results, it was determined that the patient would benefit from laparotomy. During the inspection of the peritoneal cavity and retroperitoneal spaces, enlarged right iliac lymph nodes were identified. The suspected nodes were removed and intraoperatively assessed as negative. The patient was intraoperatively qualified for Wertheim–Meigs Querleu–Morrow type B2 radical hysterectomy with ovarian transposition. In the final histology report, the cervical stump exhibited intense, chronic inflammation with foreign body-type granulomas, necrotic foci, and hemorrhages. In certain areas, normotypic squamous epithelium was preserved. No pathological changes were detected in the lymph nodes, endometrium, fallopian tubes, ovaries, or vagina.

## 12. Case Study II: Chemotherapy during Pregnancy

A 28-year-old woman with a history of two miscarriages and one term cesarean section underwent standard cervical screening in the first trimester. The patient was not vaccinated against HPV, reported no history of smoking, and had used oral contraception in the past. Chronic conditions in this patient included protein S deficiency, antithrombin III deficiency, and hypothyroidism. She had two negative Pap smears in the past—at the age of 23 and 25 years. The first positive cytology result—high-grade squamous intraepithelial lesion (HSIL)—was obtained during a routine gynecological visit one month before conception. A co-test was performed in the beginning of the pregnancy with the result of atypical glandular cells (AGCs) together with detection of HPV16.

The patient was referred to a specialist center for further diagnostic testing. Colposcopy revealed major abnormal findings: dense aceto-white epithelium located in quadrants I, II, and III, covering the whole posterior and 15% of the anterior lip of the cervix. Three biopsies were collected from the cervix with no biopsy from the cervical canal because of pregnancy. The histology result showed low-grade adenocarcinoma.

Gadolinium-enhanced pelvic MRI was performed in the 22nd gestational week. Abnormal tissue with dimensions 30 (TS) × 13 (AP) × 20 (CC) mm located in the posterior vaginal fornix in connection with the external outlet of the cervix was detected. The lesion was characterized by restricted diffusion and poor contrast enhancement. No radiologic features of uterine corpus or parametrial infiltration were described. There were no enlarged pelvic lymph nodes and no features of rectal or urinary bladder infiltration. Based on the radiological findings, the patient was diagnosed with FIGO IIA1 cervical cancer.

The patient’s wish was to continue pregnancy and at the same time be treated for the disease. However, in the first referral center, after consultation with a multidisciplinary team including a gynecologic oncologist and perinatologist, the proposed course was termination of the pregnancy followed by surgery, with no other treatment alternatives. At that moment, the patient discharged themselves from the first institution and looked for help elsewhere. Another referral center proposed a different approach—the patient was qualified for chemotherapy during pregnancy. She received four cycles of paclitaxel (175 mg/m^2^) and carboplatin (AUC5-20%) at the 24th, 27th, 31st, and 33rd weeks of gestation. Maternal and fetal well-being during chemotherapy were monitored with cardiotocography and ultrasound examinations, all of which showed normal results. The patient received psychological support offered in the center.

Cesarean delivery was scheduled for the 37th gestational week, which was four weeks after the completion of the last chemotherapy cycle. A healthy male neonate was assigned an Apgar score of 10 points, weighed 2920 g, and was 52 cm long. The delivery was uncomplicated. No abnormalities were detected during hospitalization in the neonatology unit.

Pelvic MRI repeated six weeks postpartum and ten weeks after completed chemotherapy showed no radiological signs of abnormalities in the area of the uterine cervix and no areas of restricted diffusion. The patient was qualified for surgical treatment. A type C1 Querleu–Morrow radical hysterectomy was performed ten weeks postpartum. The histology reported showed residual tissue of cervical adenocarcinoma in situ (0.7 cm in length) without signs of cervical stroma infiltration. The pathology-free margin from the vaginal cuff side was 0.9 mm. No pathologic abnormalities were detected in the ilio-obturator lymph nodes, the rest of the uterus, the tubes, the ovaries, or in the parametrial tissues. The patient received three fractions of adjuvant vaginal cuff brachytherapy, 7 Gy each. A follow-up visit was planned after 6 months.

## 13. Conclusions

Discrepancies in therapeutic options proposed among centers within the same region lead to the conclusion that there is a need for centralization and unification of evidence-based management in referral centers with both high-level oncological and perinatal care. The current guidelines for the treatment of cervical cancer in pregnant women are still inconsistent and in most countries are based on limited cohort studies. At the same time, we are gradually witnessing more published evidence leading to changes in the diagnostic approach, for example, in terms of radiological protocols for pregnant patients or use of sentinel lymph node biopsies. The choice of diagnostic procedures for pregnant patients requires a balance between the sensitivity and quality of the findings and the surgical extent or use of substances associated with a potential risk to the fetus (e.g., gadolinium-guided MRI vs. MRI without gadolinium, or conization vs. cervical biopsies).

## Figures and Tables

**Table 1:** Recommendations regarding the use of sentinel lymph node biopsy (SLNB) in cervical cancer.

Recommendation Aspect	ESGO/ESTRO/ESP Guidelines (2023) [15]	SEOM-GEICO Guidelines (2023/2024) [27]	Polish Society of Gynecological Oncology Guidelines (2024) [28]
Indication for SLNB **	T1a1: LVSI-positive patients; T1a2: SLN biopsy can be considered in LVSI-negative patients and should be performed in LVSI-positive patients; T1b1, T1b2, T2a1: SLN * biopsy should be performed before pelvic lymphadenectomy.	T1a1: LVSI-positive patients; T1a2: SLN biopsy can be considered in LVSI-negative patients and should be performed in LVSI-positive patients; T1b1, T1b2, T2a1: SLN biopsy should be performed before pelvic lymphadenectomy.	Preferred method for tumors ≤ 2 cm. May be considered for tumors >2 cm ≤4 cm.
Use in Locally Advanced Cervical Cancer (Stages IB2–IIA)	T1b1, T1b2, T2a1: SLN biopsy should be performed before pelvic lymphadenectomy.	T1b1, T1b2, T2a1: SLN mapping and any suspicious nodes should be removed intraoperatively.	T1b1/2: SLNB ** T1b3/4: PLND *** + PALND ****
Role of SLNB ** in Avoiding Complete Lymphadenectomy	T1b1, T1b2, and T2a1: After SLN biopsy, if SLN are negative on frozen section, a systematic pelvic lymphadenectomy should be performed as the standard LN staging.	T1b1, T1b2, and T2a1: If both sides reveal negative SLN in pelvic level I, LN dissection can be confined to level I. PALN dissection may be considered to reduce the risk of undetected occult metastases when imaging shows no PALN involvement.	In cases of high risk of metastasis, lymphadenectomy should include the removal of radiologically negative pelvic and para-aortic lymph nodes up to the left renal vein.
Histopathological Evaluation of SLNs	Requires ultrastaging. Intraoperative assessment should be performed on a grossly suspicious sentinel node and may be performed on a “non-suspicious” SLN.	SLNs should undergo ultrastaging to detect low-volume metastasis.	SLNs should be submitted for ultrastaging if negative H&E.

* SLN(s)—sentinel lymph nodes(s); ** SLNB—sentinel lymph node biopsy; *** PLND—pelvic lymph node dissection; **** PALND—para-aortic lymph node dissection.

## Data Availability

Not applicable.
